# Meditative and non-meditative mindfulness-based interventions for mind and body

**DOI:** 10.1186/s12906-023-04069-7

**Published:** 2023-07-15

**Authors:** Kyriaki Giannou, Michail Mantzios

**Affiliations:** 1 Independent Researcher, Birmingham, UK; 2 School of Social Sciences, Birmingham City University, Birmingham, UK

## Abstract

The present editorial synopsises the benefits and challenges in meditative and non-meditative mindfulness practices and explores shorter and more creative approaches in mind–body interventions, emphasizing inclusivity and evidence-based practices. This collection, launched in BMC Complementary Medicine and Therapies, aims to bring together research on a variety of mindful practices, to discuss their role in supporting wellbeing.

Kabat-Zinn defined mindfulness as ‘paying attention in a particular way: on purpose, in the present moment, and nonjudgmentally’ ([1] p4). This definition implies a practice and state of mindfulness that can be understood as a way of being, which can be cultivated through particular methods and techniques. Mindfulness is achieved through formal and informal practices, primarily involving meditation focused on the breath or other points of focus.

As a mind–body practice, formal mindfulness meditation entails observing the breath (or another focal point) without reacting to present-moment experiences and accepting them as they are. Mindfulness-based meditation typically requires regular practice over several weeks, which commitment and duration some people find challenging, but shorter practices have been developed to enhance mindfulness in the present moment. A surge in mindfulness and meditation research has revealed the enhancement of physical and mental health through mindfulness and meditation. For instance, mindfulness interventions have shown to enhance physical health outcomes by lowering blood pressure, improving immune functioning, and managing pain (e.g., [2]), and reducing mental health symptoms like depression, anxiety, and stress (e.g., [3]).

Formal meditative practices are often complemented by informal practices of mindfulness in daily activities like walking and eating. Mindful walking entails a focused awareness of each step, avoiding autopilot mode. Distractions are acknowledged, and attention is redirected to the sensation of walking and footfalls on the floor. Walking mindfully has been shown to reduce trait anxiety symptoms and negative affect (e.g., [4]). Other research has investigated the raisin exercise, which directs meditators to experience a raisin with all senses (e.g., texture, smell, and taste) while adopting an attentive and non-judgmental attitude. Mantzios et al. replaced the raisin with chocolate showing this practice to reduce food consumption, even in an obesogenic setting (i.e., watching a movie; [5]). Formal and informal practices together promote self-care for both mind and body.

Notably, various challenges have also emerged in this field. Grossman and Van Dam discussed the translation, adaptation, and difficulties in defining and measuring mindfulness [6]. Grossman and Van Dam further debated Western terminology and cultural appropriation, while Purser and Milillo expressed concerns about the oversimplification and commercialisation of mindfulness practices [7]. Moreover, people often oppose engaging in meditation-based practices, due to presumptions around mindfulness and meditation, or, because of potential conflicts with cultural and religious backgrounds; all in all, suggesting the need for accessible practices, which may allow for an introduction and easier uptake to mindfulness that is inexpensive and more appealing to a larger part of the population. Sharing such concerns, and with the aim to create more inclusive and creative approaches that would reach wider audiences, non-meditative practices have also been developed, expanding the range of contemplative mind–body interventions.

Non-meditative practices have emerged to achieve mindfulness benefits without the commitment of meditation. These practices target specific behaviours by incorporating mindfulness into cognitive or behavioural properties. For instance, Adams et al. developed mindfulness instructions for the process of trying on a bathing suit, which typically increases negative affect and body dissatisfaction in female participants. These instructions encouraged observation of the body in the mirror, but with an attitude of nonjudgment and acceptance. Adams et al. showed that, compared to trying on the bathing suit in silence, listening to mindfulness instructions during the process increased participants’ mindfulness levels without impacting their negative affect or body dissatisfaction [8].

Further creative approaches induced mindfulness through colouring books and embedded mindfulness instructions in the eyewitnesses’ facial composite construction process. For example, Mantzios and colleagues found that compassion meditation and guided compassion colouring had similar effects on increasing mindfulness and compassion and reducing anxiety [9]. Additionally, Giannou et al. embedded mindfulness instructions into a self-administered facial composite system, improving the construction of facial composites of famous footballers and their subsequent identification [10]. Such methods have the potential to provide a basis for introducing meditative mindfulness practices, where most of the tradition and evidence currently convenes. Therefore, research has successfully found ways of inducing the benefits of mindfulness through traditional meditative and non-meditative practices, proposing benefits for mental and physiological health, with an underlying emphasis on cognitive enhancement.

With an overall aim to support individuals’ overall health and well-being, the significance of mindfulness-based research projects rests on the exploration of a wide range of modalities in practice. This appears to be a sustainable way to support a higher uptake and commitment to mindfulness and meditation and become more considerate of individual needs and preferences. Informed by the previously mentioned challenges and criticisms, offering ways and methods that lead to traditional and rigorously tested mindfulness-based interventions are significant research avenues in moving mindfulness and meditation forward.

The collection: Mind–body interventions: mindfulness and meditation, launched in *BMC Complementary Medicine and Therapies*, welcomes research involving, but not limited to, mindful practices such as meditation, breathwork, self-compassion, relaxation, mindfulness-based interventions, guided imagery, and any non-meditative practices. Simultaneously, given the existing challenges in the field, we encourage critical evaluations of existing literature and the replication of studies to account for possible publication bias.

## Data Availability

Not applicable.
